# Effectiveness of CoronaVac in children 3–5 years of age during the SARS-CoV-2 Omicron outbreak in Chile

**DOI:** 10.1038/s41591-022-01874-4

**Published:** 2022-05-23

**Authors:** Alejandro Jara, Eduardo A. Undurraga, José R. Zubizarreta, Cecilia González, Johanna Acevedo, Alejandra Pizarro, Verónica Vergara, Mario Soto-Marchant, Rosario Gilabert, Juan Carlos Flores, Pamela Suárez, Paulina Leighton, Pablo Eguiguren, Juan Carlos Ríos, Jorge Fernandez, Heriberto García-Escorza, Rafael Araos

**Affiliations:** 1 Ministry of Health, Santiago, Chile; 2 Facultad de Matemáticas, Pontificia Universidad Católica de Chile, Santiago, Chile; 3 Center for the Discovery of Structures in Complex Data (MiDaS), Santiago, Chile; 4 Escuela de Gobierno, Pontificia Universidad Católica de Chile, Santiago, Chile; 5 Initiative for Collaborative Research in Bacterial Resistance (MICROB-R), Santiago, Chile; 6 Research Center for Integrated Disaster Risk Management (CIGIDEN), Santiago, Chile; 7 CIFAR Azrieli Global Scholars Program, Toronto ON, Canada; 8 Department of Health Care Policy, Harvard Medical School, Boston, MA USA; 9 Department of Biostatistics, Harvard T. H. Chan School of Public Health, Boston, MA USA; 10 Department of Statistics, Harvard T. H. Chan School of Public Health, Boston, MA USA; 11 Facultad de Medicina, Pontificia Universidad Católica de Chile, Santiago, Chile; 12 Instituto de Ciencias e Innovación en Medicina, Facultad de Medicina, Clínica Alemana Universidad del Desarrollo, Santiago, Chile; 13 Advanced Center for Chronic Diseases (ACCDiS), Santiago, Chile

**Keywords:** Epidemiology, Viral infection

## Abstract

The outbreak of the B.1.1.529 lineage of severe acute respiratory syndrome coronavirus 2 (SARS-CoV-2) (Omicron) has caused an unprecedented number of Coronavirus Disease 2019 (COVID-19) cases, including pediatric hospital admissions. Policymakers urgently need evidence of vaccine effectiveness in children to balance the costs and benefits of vaccination campaigns, but, to date, the evidence is sparse. Leveraging a population-based cohort in Chile of 490,694 children aged 3–5 years, we estimated the effectiveness of administering a two-dose schedule, 28 days apart, of Sinovac’s inactivated SARS-CoV-2 vaccine (CoronaVac). We used inverse probability-weighted survival regression models to estimate hazard ratios of symptomatic COVID-19, hospitalization and admission to an intensive care unit (ICU) for children with complete immunization over non-vaccination, accounting for time-varying vaccination exposure and relevant confounders. The study was conducted between 6 December 2021 and 26 February 2022, during the Omicron outbreak in Chile. The estimated vaccine effectiveness was 38.2% (95% confidence interval (CI), 36.5–39.9) against symptomatic COVID-19, 64.6% (95% CI, 49.6–75.2) against hospitalization and 69.0% (95% CI, 18.6–88.2) against ICU admission. The effectiveness against symptomatic COVID-19 was modest; however, protection against severe disease was high. These results support vaccination of children aged 3–5 years to prevent severe illness and associated complications and highlight the importance of maintaining layered protections against SARS-CoV-2 infection.

## Abstract (web summary)

CoronaVac protects young children from severe COVID-19 during a SARS-CoV-2 Omicron surge, supporting the effectiveness and importance of vaccinating this pediatric population.

## Main

The emergence and spread of the B.1.1.529 lineage of SARS-CoV-2, the cause of COVID-19, has caused an unprecedented number of infections worldwide in a short period^1,2^. Emerging evidence suggests that Omicron causes less severe disease than previous variants of concern (VOCs), probably due to lower virulence, infection-acquired immunity and higher vaccination coverage^3–6^. However, its high transmissibility and ability to partially evade the immune response induced has been associated with a substantial increase in severe COVID-19 cases globally^2^. The absolute number of pediatric hospital admissions has also surpassed previous waves^4,7,8^, straining healthcare systems even further. The increase may be related to higher transmissibility of Omicron, less use of face masks in children and, especially concerning, lower vaccination rates among children.

Policymakers urgently need evidence of the effectiveness of vaccines in preventing severe clinical presentations of COVID-19 in children to balance the costs and benefits of mass vaccination campaigns in this population. Although the risk of severe COVID-19 in healthy children is substantially lower than among adults, vaccinating children may reduce community transmission, avoid potentially life-threatening presentations such as multisystemic inflammatory syndrome in children (MIS-C) or pediatric inflammatory multisystem syndrome (PIMS) and prevent long-term consequences of SARS-CoV-2 infection^9^. Although many countries are vaccinating children, few have authorized COVID-19 vaccines for children under 5 years of age, and some have restricted vaccines for children older than 12 years^10^. Evidence of the efficacy or effectiveness of COVID-19 vaccines in children is limited, primarily related to mRNA vaccines, and only two studies were conducted during the Omicron outbreak^11–15^. To our knowledge, there is no published evidence of vaccine effectiveness against COVID-19 in young children under 5 years of age. Furthermore, recent research suggests that several COVID-19 vaccine platforms provide limited protection against infection and symptomatic disease caused by the Omicron variant but were more effective against severe disease^16–18^. These studies have examined vaccine protection against Omicron in adult populations but are consistent with preliminary, unpublished results from a study in children aged 5–12 years^13^.

Leveraging a population-based cohort of children aged 3–5 years, we estimated the effectiveness of the complete primary immunization schedule (two doses, 28 days apart) of an inactivated SARS-CoV-2 vaccine, CoronaVac, to prevent laboratory-confirmed, symptomatic COVID-19, hospitalization and admission to an ICU. We estimated vaccine effectiveness using inverse probability-weighted survival regression models to estimate hazard ratios of complete immunization (starting 14 days after the second dose) over the unvaccinated status, accounting for time-varying vaccination exposure and available clinical, demographic and socioeconomic confounders at baseline.

**Fig. 1:**
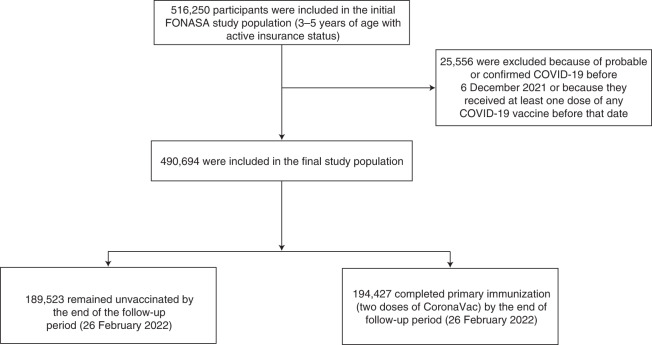
Study participants and cohort eligibility, 6 December 2021 through 26 February 2022. Participants were 3–5 years of age, affiliated to the FONASA, the public national healthcare system, and received two doses of CoronaVac, 28 days apart between 6 December 2021 and 26 February 2022 or did not receive any COVID-19 vaccination. We excluded children who had probable or confirmed COVID-19 according to RT–PCR assay for SARS-CoV-2 or antigen test before 6 December 2021.

**Extended Data Fig. 1:**
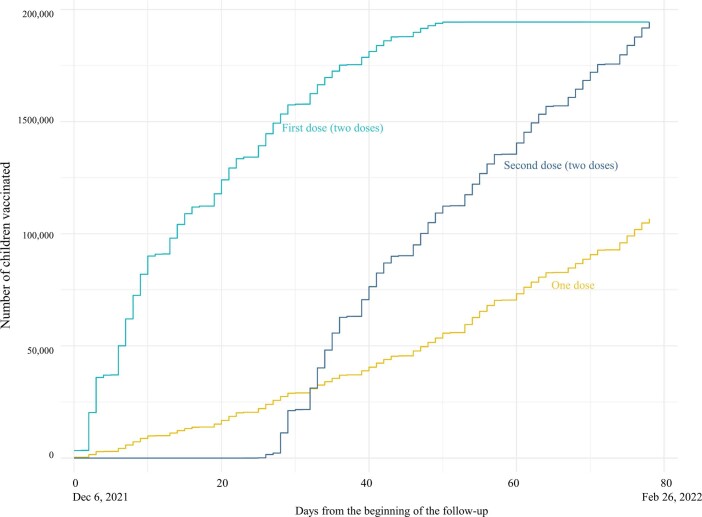
CoronaVac vaccination rollout among children aged 3 to 5 years, by vaccination group. unvaccinated, vaccinated with one dose, vaccinated with two doses after 28 days. The Public Health Institute of Chile extended the authorization for emergency use of CoronaVac to children starting at three years of age on November 25, 2021. The first children aged 3-5 years were vaccinated on December 6, 2021, prioritizing immunocompromised children and those with comorbidities. The median date of first and second dose for all children in the cohort were 14 and 50 days from the beginning of the follow-up respectively.

**Extended Data Fig. 2:**
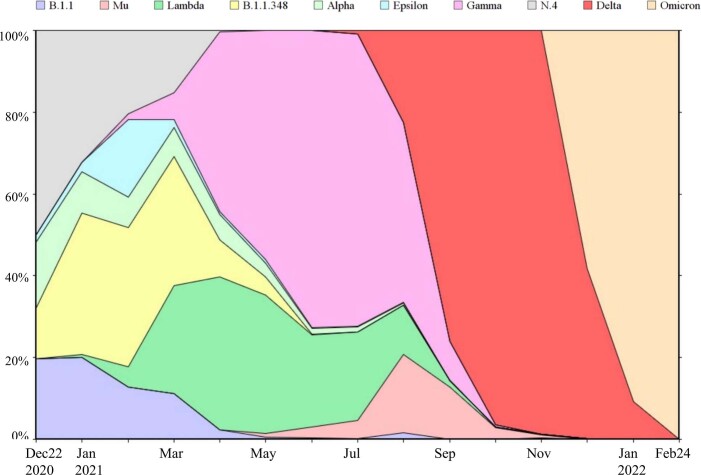
Evolution of the predominant SARS- CoV-2 lineages in Chile, according to data shared on GISAID platform, December 22, 2020, to February 24, 2022. The Ministry of Health monitors respiratory viruses, including SARS- CoV-2, using genomic surveillance in sentinel centers. Surveillance uses non-probabilistic sampling of SARS-CoV-2 infections focusing on variants of concern (VOC) and variants of interest (VOI) through traveler (imported cases) and community surveillance (hospitalized cases and national core priority studies). The samples are sent for whole-genome sequencing (WGS) and genotyping across the country. Between December 22, 2020, and February 21, 2022, 70,186 SARS-CoV-2 samples were analyzed. Of these, 28.6% (n=20,088) were sequenced and 71.4% (n=50,098) assessed by detection of variant-associated mutations (VAM) using RT- PCR. Of these analyzed samples, 85.3% (n=59,891) correspond to VOC and 5.2% (n=3,618) to variants of interest (VOI).

Our study cohort included 516,250 children aged 3–5 years affiliated with the Fondo Nacional de Salud (FONASA), the public national healthcare system of Chile. In total, 490,694 children were included in the final study population; 194,427 had received two doses of CoronaVac, 28 days apart between 6 December 2021 and 26 February 2022; and 189,523 had not received any COVID-19 vaccination by the end of the follow-up period. On 25 November 2021, the Public Health Institute of Chile authorized the emergency use of CoronaVac on young children (3–5 years) and began vaccinating on 6 December 2021. CoronaVac was the only COVID-19 vaccine authorized for young children during the study period. We excluded children who had probable or confirmed COVID-19 according to reverse transcription polymerase chain reaction (RT–PCR) assay for SARS-CoV-2 or antigen test before 6 December 2021, reported to the Ministry of Health (Fig. 1). The cohort characteristics are described in Extended Data Tables 1 and 2. We found statistically significant differences (*P* < 0.001) in the incidence of COVID-19 and according to vaccination status by children’s sex, age group, comorbidities, nationality, region of residence and insurance category, which justify their inclusion in the models. Vaccination rollout was organized through a public schedule; children needed to show up at their nearest vaccination site with their national ID card (Extended Data Fig. 1). The study period overlapped with that of the Omicron outbreak in Chile (with the Omicron BA.1.1 sublineage predominant), defined by whole-genome sequencing of a sample of the infecting variants circulating over time (Extended Data Tables 3–5 and Extended Data Fig. 2).

The estimated adjusted vaccine effectiveness for CoronaVac in children aged 3–5 years during the Omicron outbreak was 38.2% (95% CI, 36.5–39.9) for the prevention of COVID-19, 64.6% (95% CI, 49.6–75.2) for the prevention of hospitalization and 69.0% (95% CI, 18.6–88.2) for the prevention of COVID-19-related ICU admission (Table 1). We did not estimate vaccine effectiveness against fatal outcomes because only two deaths were observed in the unvaccinated group as of 26 February 2022, the study end.

**Table 1:** Effectiveness of the CoronaVac COVID-19 vaccine in preventing symptomatic COVID-19 outcomes in children 3–5 years of age in the study cohort according to immunization status, 6 December 2021 through 26 February 2022^a^

Immunization status		Cases		Vaccine effectiveness (95% CI)	
	Person-days	No.	Incidence rate per 1,000 person-days	Weighted, standard adjustment^b^	Weighted, stratified analysis^b^
**Symptomatic COVID-19**					
Unvaccinated	29,404,535	7,555	0.2569	–	–
CoronaVac	18,499,492	4,562	0.2466	37.9	38.2
(≥14 days after second dose)	(36.1; 39.6)	(36.5; 39.9)			
**Hospitalization**					
Unvaccinated	29,579,595	62	0.0021	–	–
CoronaVac	18,990,209	23	0.0012	65.2	64.6
(≥14 days after second dose)	(50.4; 75.6)	(49.6; 75.2)			
**Admission to ICU**					
Unvaccinated	29,580,825	9	0.0003	–	–
CoronaVac	18,993,888	3	0.0002	68.8	69.0
(≥14 days after second dose)	(18.0; 88.1)	(18.6; 88.2)			

^a^We classified participants’ status into two categories during the study period: unvaccinated and fully immunized (≥14 days after receiving the second dose of CoronaVac). The days between the first dose vaccine administration and the full immunization were excluded from the at-risk person-time. We provide the results for the standard and stratified versions of the Cox hazard models using inverse probability of treatment weighting.

^b^The analyses were adjusted for age, sex, region of residence, health insurance category (a proxy of household income), nationality and whether the patient had underlying conditions that have been associated with severe COVID-19 in children, coded as described in Supplementary Table 1. The standard and stratified versions of the extended Cox proportional hazard models were fit to test the robustness of the estimates to model assumptions.

**Extended Data Fig. 3:**
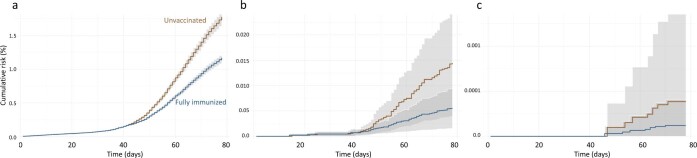
Extended Fig.3 Estimated cumulative incidence of (a) symptomatic COVID- 19, (b) hospitalization, and (c) admission to incentive care unit (ICU) for unvaccinated and fully immunized individuals. Comparison of the cumulative incidence curves between unvaccinated and fully immunized children (≥ 14 days after receiving the second dose of the CoronaVac COVID-19 vaccine) on January 1, 2022. The estimates are presented as the mean values, with 95% point-wise confidence intervals, for boys, aged 4, affiliated to FONASA insurance type A, and not having comorbidities.

Our estimates provide evidence of vaccination effectiveness in children aged 3–5 years during the Omicron outbreak in Chile (Table 1 and Extended Data Fig. 3). These results are substantially lower than recent preliminary estimates of the effectiveness of two-dose vaccination of CoronaVac in children 6–16 years, in a period when B.1.617.2 (Delta) was the predominant circulating SARS-CoV-2 variant^14^. In that study, the estimated effectiveness in children 6–16 years was 74.5% (95% CI, 73.8–75.2) for the prevention of COVID-19, 91.0% (95% CI, 87.8–93.4) for the prevention of hospitalization and 93.8% (95% CI, 87.8–93.4) for the prevention of COVID-19-related ICU admission. The estimates for the subgroup of children aged 6–11 years were 75.8% (95% CI, 74.7–76.8) for the prevention of COVID-19 and 77.9% (95% CI, 61.5–87.3) for the prevention of hospitalization^14^. Although the estimates are not directly comparable, the lower estimated vaccine effectiveness in this study could be due to Omicron or because the cohort included younger children. Vaccine effectiveness was estimated shortly after vaccination. In light of recent evidence suggesting that the effectiveness of a two-dose COVID-19 vaccination against infection and symptomatic disease wanes over time^19^, our estimates of protection for children aged 3–5 years may be at their highest level.

Recent research suggests that currently available vaccines may be less effective against Omicron. Consistent with our results, an unpublished study in New York^13^ found that the effectiveness of two BNT162b2 vaccine doses for the prevention of COVID-19 and hospitalization decreased from 66% to 51% and from 85% to 73% for children aged 12–17 years, respectively. The drop was more considerable among children aged 5–11 years; protection against COVID-19 fell from 68% to 12%; and protection against hospitalization fell from 100% to 48%^13^. Preliminary, unpublished results from a large cohort of children aged 3–11 years in Argentina show that two doses of Sinopharm’s inactivated SARS-CoV-2 vaccine BBIBP-CorV were 59% effective against hospitalization when Omicron was the predominant variant and 83% effective when Delta and Omicron circulated^15^. Results among adults tell the same story. Early data from South Africa reported that BNT162b2 protection against COVID-19-related hospitalization decreased from 93% to 70% among adults^16^. Among adults in the United Kingdom, two doses of ChAdOx1 nCoV-19 provided no detectable protection against the Omicron variant after 20 weeks, and two doses of BNT162b2 were only 8.8% effective against Omicron after 25 weeks^17^. The study suggests that a BNT162b2 or mRNA-1273 booster substantially increased protection against Omicron^17^. Similarly, a study that evaluated serum neutralization against Omicron or the D614G variant among adult participants with the mRNA-1273 vaccine primary series observed neutralization titers 35 times lower for Omicron^18^.

Children’s age could also potentially affect vaccine effectiveness estimates for severe disease, as suggested by older children in recent unpublished studies in New York^13^ and Chile^14^. Clinical trials for Moderna’s mRNA-1273 and Pfizer-BioNTech’s BNT162b2 in children 6 months of age to under 5 years of age are being conducted. Preliminary results for two 3-µg doses, 21 days apart, of the BNT162b2 in children 2 years of age to under 5 years of age did not produce an adequate immune response, although the immune response of children between 6 months of age and 2 years of age was similar to that of young adults^20^. Data from the mRNA-1273 vaccine in children have not yet been released.

Observational studies have limitations. Selection bias could affect vaccine effectiveness estimates if the vaccinated and unvaccinated groups are systematically different. We partially addressed this issue by adjusting our estimates with observable confounders that may affect vaccination and the risk of COVID-19. However, we do not have data to assess whether vaccinated and unvaccinated children or their caregivers differ in some unobservable characteristics, such as compliance with COVID-19 behavioral guidelines. Another limitation in our study relates to genomic surveillance capabilities. The Ministry of Health’s strategy has focused on detecting VOCs through traveler and community surveillance but uses non-probabilistic sampling (Extended Data Fig. 2 and Extended Data Tables 3–5). There were few child admissions to the ICU associated with SARS-CoV-2 infection during our study period, which led to wide CIs in our estimates. Finally, because laboratory-confirmed COVID-19 cases depend on the patients’ healthcare-seeking behavior, it is possible that asymptomatic or mildly symptomatic cases were missed in our study. Although this may occur in both groups, immunized children may be more likely to develop mild symptoms due to vaccine-induced protection than unvaccinated children. If so, we might have overestimated protection against symptomatic infection. However, this potential bias would not have affected our effectiveness estimates for protection against COVID-19-related hospitalization and ICU admission. Our study examined the effectiveness of a two-dose CoronaVac schedule; the results may be different with a homologous or heterologous booster dose, as shown for adults.

Strengths of the study include that data were collected during the Omicron outbreak, with the highest transmission rates since the beginning of the pandemic. Vaccination rollout in Chile was quick and had high uptake (Extended Data Fig. 1). Our estimated vaccine effectiveness reflects a ‘real-life’ situation by including the challenges public health officials face in the field, such as a more diverse set of participants (for example, with underlying conditions), schedule compliance, logistics and cold chains. These estimates may be essential for decision-making as a complement to controlled clinical trials.

Overall, our results show that the effectiveness of a complete primary immunization schedule with CoronaVac in children 3–5 years of age against symptomatic COVID-19 during the Omicron outbreak was limited. However, vaccines were effective against severe disease in young children. These results support the vaccination of children 3–5 years of age to prevent severe illness and associated complications; however, they underscore the importance of maintaining layered protections against SARS-CoV-2 infection in this population. Important next steps include examining how long vaccine protection lasts and whether booster shots will be necessary. We hope that the results from this study inform policymakers in countries considering child vaccination against COVID-19.

## Methods

### Outcomes

The Ministry of Health in Chile requires that all suspected COVID-19 cases are immediately notified to health authorities through Epivigila, an online platform that centralizes all case notification and test results and represents the case count source used for this study. Suspected COVID-19 cases require laboratory testing with RT–PCR assay or antigen tests. We estimated the vaccine effectiveness of CoronaVac for children aged 3–5 years using three primary outcomes: laboratory-confirmed symptomatic SARS-CoV-2 infection (COVID-19), hospitalization and admission to the ICU associated with SARS-CoV-2 infection. We considered the time to the onset of symptoms from the beginning of the follow-up, 6 December 2021, as the endpoint of each outcome. We used the onset of symptoms as a proxy for the time of infection. We classified participants’ status into two categories along the study period: unvaccinated and fully immunized (≥14 days after receipt of the second dose with CoronaVac). A child was excluded from the unvaccinated group when she or he received the first vaccine dose. The period between the first dose administration and 13 days after the second dose was excluded from the at-risk person-time in our analyses.

### Statistical analyses

We used Bonferroni-adjusted Pearson’s χ^2^ to compare descriptive data and control for multiple comparisons. To estimate hazard ratios, we used extensions of the Cox hazard model that allowed us to account for the time-varying vaccination status of participants^21–23^. We adjusted for differences in observed individual characteristics by inverse probability of treatment weighting as in marginal structural models^24^, estimating the weights non-parametrically^25^. Vaccine effectiveness was estimated based on the hazard ratio between the treated and non-treated status. We reported hazard ratio estimates adjusted for age, sex, region of residence, nationality, health insurance category (a proxy of household income) and underlying conditions (Extended Data Tables 1 and 2) under the standard and stratified versions of the Cox hazard model.

Let *T*_*i*_ be the time-to-event of interest, from 6 December 2021, for the *i*-th individual in the cohort, $i = 1, \ldots ,n$. Let $x_i,i = 1, \ldots ,n$ be a *p-*dimensional vector of individual-specific characteristics, such as age and sex, and let *z*_*i*_(*t*) be the time-dependent treatment indicator. The model assumes that the time-to-events are independent and with probability distribution given by

$$T_i|x_i,z_i\sim f\left( {t|x_i,z_i} \right),i = 1, \ldots ,n,$$

where

$$\begin{array}{l}f\left( {t|x_i,z_i} \right) = \lambda _0\left( t \right)exp\left\{ {x_i^\prime \gamma + \beta _{z_i(t)}} \right\} \times \\ exp\left\{ { - exp\left\{ { - x_i^\prime \gamma + \beta _{z_i(t)}} \right\}\mathop {\int}\limits_0^t {\lambda _0} \left( u \right)du} \right\},\end{array}$$

with $\gamma \in {\Bbb R}^p$ being a vector of regression coefficients, $\beta _k \in {\Bbb R}$ being the regression coefficient measuring the effectiveness of the *k*^*th*^ vaccine and λ_0_ being the baseline hazard function

$$\lambda _0\left( t \right) = \mathop {{\lim }}\limits_{h \to 0} \left\{ {\frac{{P_0\left( {t \le T \le t + h|T \ge t} \right)}}{h}} \right\},$$

where *P*_0_ is the baseline probability distribution. A Cox model with time-dependent covariates compares the risk of the event of interest between immunized and non-immunized participants at each event time but re-evaluates which risk group each person belonged to, based on whether they had been immunized by that time.

We also fitted a stratified version of the model^26^, where the time-to-event distribution is given by

$$\begin{array}{l}f\left( {t|x_i,z_i} \right) = \lambda _{x_i,0}\left( t \right)exp\left\{ {\beta _{z_i(t)}} \right\} \times \\ exp\left\{ { - exp\left\{ {\beta _{z_i(t)}} \right\}\mathop {\int}\limits_0^t {\lambda _{x_i,0}} \left( u \right)du} \right\},\end{array}$$

with $\beta _k \in {\Bbb R}$ being the regression coefficient measuring the effectiveness of the *k*th vaccine, and λ_*x*,0_ is the predictor-specific baseline hazard function. We fitted a stratified version of the extended Cox proportional hazard model to test the robustness of our estimates to model assumptions. Under the stratified Cox model, each combination of predictors has a specific hazard function that can evolve independently.

We estimated the vaccine effectiveness as $100\% \cdot \left( {1 - exp\left\{ {\beta _k} \right\}} \right)$. We show the adjusted vaccine effectiveness results, including covariates as controls (age, gender, region, nationality, health insurance category and comorbidities). We show the results for the standard and stratified versions of the Cox hazard model using inverse probability of treatment weighting. We computed standard 95% Wald CIs for the estimates. Inference was based on a partial likelihood approach^27^. Recall that the effectiveness estimate for the COVID-19 vaccines in the Cox model with time-dependent vaccination status compares the risk of an event for children who received the vaccine and those who were unvaccinated at each event time. The risk group is determined by whether the child had received the vaccine shot or not in a specific calendar time, and the comparison of the risk of an event is made at the same calendar time. Each term in the partial likelihood of the effectiveness regression coefficient corresponds to the conditional probability of an individual to express the outcome of interest from the risk set at a given calendar time.

Under the standard Cox model, all individuals at risk are included in the risk set, and their contribution is weighted based on their covariates (as shown in Extended Data Table 1). Under the stratified version of the Cox model, each stratum has a different risk set determined by the covariates.

We conducted the analysis with the survival package^28^ of R, version 4.0.5 (ref. ^29^).

### Ethics statement

The research protocol was approved by the Comité Ético Científico Clínica Alemana Universidad del Desarrollo (Santiago, Chile). No human health risks as a result of our study were identified because we analyzed administrative datasets. The study was considered exempt from informed consent.

### Reporting summary

Further information on research design is available in the Nature Research Reporting Summary linked to this article.

## Online content

Any methods, additional references, Nature Research reporting summaries, source data, extended data, supplementary information, acknowledgements, peer review information; details of author contributions and competing interests; and statements of data and code availability are available at 10.1038/s41591-022-01874-4.

## Supplementary information

Supplementary Information

Reporting Summary

## Data Availability

Owing to data privacy regulations, the individual-level data used in this study cannot be shared (Law N19.628). Aggregate data on vaccination and COVID-19 incidence are publicly available at https://github.com/MinCiencia/Datos-COVID19/.

## Extended data

**Extended Data Table 1:** Characteristics of the study cohort of children affiliated to FONASA, overall, with laboratory-confirmed COVID-19 and the proportion receiving one or more doses of COVID-19 vaccines, 6 December 2021 through 26 February 2022^a^

							Vaccinated			
			COVID-19		Unvaccinated		One dose		Two doses	
Characteristic	No.	Col. %	No.	Row %	No.	Row %	No.	Row %	No.	Row %
Total	490,694	100	14,512	3.0	189,523	38.6	106,744	21.8	194,427	39.6
**Sex**										
Female	241,429	49	6,815	2.8	90,586	38	52,593	22	98,250	41
Male	249,265	51	7,697	3.1	98,937	40	54,151	22	96,177	39
**Cohort location**										
Arica	7,488	1.5	304	4.1	3,739	50	1,774	24	1,975	26
Tarapacá	11,165	2.3	259	2.3	5,407	48	2,592	23	3,166	28
Antofagasta	16,652	3.4	350	2.1	6,440	39	3,937	24	6,275	38
Atacama	8,687	1.8	409	4.7	3,514	40	2,118	24	3,055	35
Coquimbo	24,079	4.9	733	3.0	9,909	41	5,578	23	8,592	36
Valparaíso	49,595	10.0	1,464	3.0	18,427	37	10,662	21	20,506	41
Metropolitana	181,781	37.0	3,925	2.2	67,537	37	38,348	21	75,896	42
LB O’Higgins	27,870	5.7	757	2.7	8,518	31	5,820	21	13,532	49
Maule	33,352	6.8	1,272	3.8	10,091	30	7,409	21	15,852	47
Ñuble	14,040	2.9	663	4.7	4,909	35	3,223	22	5,908	42
Biobío	43,107	8.8	1,857	4.3	16,564	38	10,090	23	16,453	38
Araucanía	31,150	6.3	951	3.1	14,083	45	6,460	23	10,607	34
Los Ríos	10,837	2.2	513	4.7	4,894	45	2,332	21	3,611	33
Los Lagos	24,781	5.1	777	3.1	12,878	52	5,026	22	6,877	28
Aysén	2,427	0.5	119	4.9	1,073	44	549	23	805	33
Magallanes	3,683	0.7	159	4.3	1,540	42	826	22	1,317	36
**Age group**										
3 years	161,379	33	4,816	3.0	76,259	47	33,096	21	52,024	32
4 years	160,829	33	4,581	2.8	60,282	37	35,919	22	64,628	40
5 years	168,486	34	5,115	3.0	52,982	31	37,729	22	77,775	46
**Comorbidities** ^b^										
None	445,074	90.7	12,669	2.8	174,187	39	96,221	22	174,666	39
≥1	45,620	9.3	1,843	4.0	15,336	34	10,523	23	19,761	43
**Nationality**										
Chilean	484,715	98.8	14,404	3.0	186,988	39	105,613	22	192,114	39.6
Non-Chilean	5,979	1.2	108	1.8	2,535	42	1,131	19	2,313	38.7

^a^On 6 September 2021, the Public Health Institute of Chile authorized the emergency use of CoronaVac for children aged 6 years and older and, on 25 November 2021, extended the age range to children starting at 3 years of age. The first children aged 3–5 years were vaccinated on 6 December 2021, prioritizing immunocompromised children and those with comorbidities, including chronic kidney disease, diabetes mellitus types 1 and 2, cancer, congenital heart disease and HIV. Our study cohort included children 3–5 years of age affiliated to FONASA, the national public health insurance program in Chile. We excluded children with probable or confirmed SARS-CoV-2 infection before 6 December 2021. The model also included health insurance category (a proxy of household income) and location (16 regions). We found statistically significant differences (*P* < 0.001) between patients with COVID-19 and the vaccinated and unvaccinated groups by sex, age group, comorbidities, nationality, region of residence and health insurance category.

^b^Coexisting conditions included chronic kidney disease, diabetes mellitus types 1 and 2, cancer, congenital heart disease, HIV, epilepsy, hemophilia, asthma, cystic fibrosis, juvenile idiopathic arthritis and systemic lupus erythematosus (see also Supplementary Table 2).

**Extended Data Table 2:** Underlying conditions associated with severe COVID-19 illness in the study cohort of children aged 3–5 years affiliated to FONASA^a^

Comorbidity	Number of cases	
	Female	Male
Total participants	241,429	249,265
Chronic kidney disease	5	3
Congenital heart disease	3,168	3,230
Cancer	318	385
Diabetes mellitus types 1 and 2	114	144
HIV	534	575
Epilepsy	935	1,082
Hemophilia	68	154
Asthma	15,172	21,641
Cystic fibrosis	24	28
Juvenile idiopathic arthritis	34	12
Systemic lupus erythematosus	0	0

^a^The study cohort included children 3–5 years of age affiliated to FONASA. We excluded children who had probable or confirmed COVID-19 according to RT–PCR assay for SARS-CoV-2 or antigen test before 6 December 2021.

**Extended Data Table 3:** Main SARS-CoV-2 variants and lineages detected in Chile through genomic surveillance, by detection method, 22 December 2020 through 21 February 2022

Variant	Genomic sequencing	VAM	Total	Proportion (%)	Subtotal	Proportion (%)
**Variants of concern**						
Alpha (B.1.1.7)	293	196	489	0.7	59,891	85.3
Beta (B.1.135)	4	1	5	0.0		
Gamma (P.1)	4,360	2,613	6,973	9.9		
Delta (B.1.617.2)	7,666	32,787	40,453	57.6		
Omicron (BA.1, BA.1.1, BA.2)	4,211	7,760	11,971	17.1		
**Variants of interest**						
Lambda (C37)	1,704	25	1,729	2.5	3,618	5.2
Mu (B.1.621)	849	1,040	1,889	2.7		
**Lineages and other varia**nts						
Other lineages^a^	1001	34	1,035	1.5	1,035	1.5
**Indeterminate**	0	5,642	5,642	8.0	5,642	8.0
**Total**	20,088	50,098	70,186	100	70,186	100

VAM denotes mutation associated with variant according to RT–PCR assay for SARS-CoV-2.

^a^Corresponds to other low-frequency lineages and unspecified variants. Preliminary data are in the process of validation.

Source: Department of Epidemiology, Ministry of Health, Chile.

**Extended Data Table 4:** Proportion of Omicron from all SARS-CoV-2 variants detected in Chile through genomic surveillance from 12 December 2021 to 19 February 2022

Epidemiological week	Omicron	Proportion	Total
	No.	%	No.
**Year 2021**			
Dec 12–18	211	13.2	1,594
Dec 19–25	711	39.5	1,798
Dec 26–Jan 1	1,746	58.6	2,980
**Year 2022**			
Jan 2–8	1,908	69.5	2,746
Jan 9–15	1,993	80.8	2,467
Jan 16–22	1,655	79.1	2,092
Jan 23–29	1,475	87.7	1,682
Jan 30–Feb 5	949	97.5	973
Feb 6–12	946	96.2	983
Feb 13–19	323	89.7	360
**Total**	11,917	67.4	17,675

No. denotes number of cases.

Source: Department of Epidemiology, Ministry of Health, Chile

**Extended Data Table 5:** Main Omicron sublineages detected through genomic sequencing in Chile during the study period

Variant of concern	Dec 6–31 2021		Jan 1–Feb 28, 2022		Total
	*n*	Proportion (%)	*n*	Proportion (%)	
Delta (B.1.617.2)	995	39.2	159	5.7	1,154
Omicron	706	27.8	1,660	59.5	2,366
BA.1	70		49		
BA.1.1	636		1,608		
BA.2	0		3		
Unassigned					
BA.1 sublineages	810	31.9	871	31.2	1,681
BA.2 sublineages	0		40	1.4	40
Other lineages	30	1.2	60	2.2	90
Total	2,541		2,790		5,331

Source: Department of Epidemiology, Ministry of Health, Chile
