# Supplementary material for: Effectiveness of CoronaVac in children 3–5 years of age during the SARS-CoV-2 Omicron outbreak in Chile
Source: Nat Med. 2022 May 23;28(7):1377–80. doi: 10.1038/s41591-022-01874-4 (PMC9307483; doi:10.1038/s41591-022-01874-4)
Supplement: Supplementary file 1 — Supplementary Information [file 41591_2022_1874_MOESM1_ESM.pdf]

nature portfolio

https://doi.org/10.1038/s41591-022-01874-4

Supplementary information

Effectiveness of CoronaVac in children 3–5 years of age during the SARS-CoV-2 Omicron outbreak in Chile

In the format provided by the authors and unedited

Effectiveness of CoronaVac in children 3 to 5 years during the omicron SARS-CoV-2 outbreak

Supplementary Information

Adverse events

The Public Health Institute of Chile is in charge of pharmacovigilance in Chile. Healthcare facilities and providers must notify of all adverse events potentially associated with Covid-19 vaccines. These are evaluated by an expert committee, including specialists from the National Pharmacovigilance Center, the Immunization National Plan (PNI, acronym in Spanish), and external clinicians. Priority is given to cases classified as serious, unexpected, and of clinical interest.

Between December 6, 2021, and February 26, 2022, the Ministry of Health administered 717.515 doses of CoronaVac vaccine to children aged 3-5 years.[1] The Chilean Institute of Public Health received 65 notifications of adverse events potentially associated with Covid-19 vaccination;[2] about 9.1 notifications per 100,000 vaccine doses administered. Of these events, 7 were classified as serious, equivalent to 1.0 event per 100,000 doses administered.

Adverse events potentially associated with vaccination may include more than one sign or symptom. The most frequent clinical manifestations were local reactions at the site of injection (3.9 per 100,000 doses). Other non-serious events reported were fever and hives (1.8 per 100,000 doses), vomit (1.5 per 100,000 doses), pruritus (1.3 per 100,000 doses), and erythema (1.1 per 100,000 doses). The most serious adverse event associated with vaccination were convulsions (0.4 per 100,000 doses). There were also reports of epilepsy, thrombocytopenic purpura, anaphylactic reaction, tachycardia, tachypnea, and thrombocytopenia, with a rate of 0.1 events per 100,000 doses administered. The adverse clinical manifestations associated with Covid-19 vaccines in children are consistent with those detected in the post-authorization period by international regulatory agencies.

References

1 Ministerio de Salud. *Departamento de Estadísticas e Información de Salud, Cifras oficiales Covid-19 en Chile*, <https://deis.minsal.cl/> (2022).

2 Instituto de Salud Pública de Chile. Base de datos RAM-ESAVI. (ISP, Santiago, Chile, 2022).

Correspondence to Dr. Rafael Araos, rafaelaraos@udd.cl
