# Supplementary material for: Effectiveness of CoronaVac in children 3–5 years of age during the SARS-CoV-2 Omicron outbreak in Chile
Source: Nat Med. 2022 May 23;28(7):1377–80. doi: 10.1038/s41591-022-01874-4 (PMC9307483; doi:10.1038/s41591-022-01874-4)
Supplement: Supplementary file 2 — Reporting Summary [file 41591_2022_1874_MOESM2_ESM.pdf]

nature portfolio

Corresponding author(s): Rafael Araos

Last updated by author(s): May 17, 2022

Reporting Summary

Nature Portfolio wishes to improve the reproducibility of the work that we publish. This form provides structure for consistency and transparency in reporting. For further information on Nature Portfolio policies, see our Editorial Policies and the Editorial Policy Checklist.

Statistics

For all statistical analyses, confirm that the following items are present in the figure legend, table legend, main text, or Methods section.

n/a	Confirmed	
☐	☒	The exact sample size (*n*) for each experimental group/condition, given as a discrete number and unit of measurement
☐	☒	A statement on whether measurements were taken from distinct samples or whether the same sample was measured repeatedly
☐	☒	The statistical test(s) used AND whether they are one- or two-sided<br>*Only common tests should be described solely by name; describe more complex techniques in the Methods section.*
☐	☒	A description of all covariates tested
☐	☒	A description of any assumptions or corrections, such as tests of normality and adjustment for multiple comparisons
☐	☒	A full description of the statistical parameters including central tendency (e.g. means) or other basic estimates (e.g. regression coefficient) AND variation (e.g. standard deviation) or associated estimates of uncertainty (e.g. confidence intervals)
☐	☒	For null hypothesis testing, the test statistic (e.g. F, t, r) with confidence intervals, effect sizes, degrees of freedom and P value noted<br>*Give P values as exact values whenever suitable.*
☒	☐	For Bayesian analysis, information on the choice of priors and Markov chain Monte Carlo settings
☐	☒	For hierarchical and complex designs, identification of the appropriate level for tests and full reporting of outcomes
☒	☐	Estimates of effect sizes (e.g. Cohen's *d*, Pearson's *r*), indicating how they were calculated

Our web collection on statistics for biologists contains articles on many of the points above.

Software and code

Policy information about availability of computer code

Data collection	R version 4.0.5
Data analysis	We conducted the analysis with the survival package of R, version 4.0.5.

For manuscripts utilizing custom algorithms or software that are central to the research but not yet described in published literature, software must be made available to editors and reviewers. We strongly encourage code deposition in a community repository (e.g. GitHub). See the Nature Portfolio guidelines for submitting code & software for further information.

Data

Policy information about availability of data

All manuscripts must include a data availability statement. This statement should provide the following information, where applicable:

- Accession codes, unique identifiers, or web links for publicly available datasets
- A description of any restrictions on data availability
- For clinical datasets or third party data, please ensure that the statement adheres to our policy

Owing to data privacy regulations, this study's individual-level data used in this study cannot be shared (Law N19.628). Aggregate data on vaccination and Covid-19 incidence are publicly available at https://github.com/MinCiencia/Datos-COVID19/.

Field-specific reporting

Please select the one below that is the best fit for your research. If you are not sure, read the appropriate sections before making your selection.

☒ Life sciences ☐ Behavioural & social sciences ☐ Ecological, evolutionary & environmental sciences

For a reference copy of the document with all sections, see nature.com/documents/nr-reporting-summary-flat.pdf

Life sciences study design

All studies must disclose on these points even when the disclosure is negative.

Sample size	We did not estimate sample size as we used a population-based cohort including all individuals affiliated with FONASA (Fondo Nacional de Salud), which include ~80% of the Chilean population.
Data exclusions	We excluded children who had probable or confirmed Covid-19 according to reverse-transcription polymerase-chain-reaction assay for SARS-CoV-2 or antigen test before December 6, 2021.
Replication	Does not apply, however, we used the survival package of R, version 4.0.5.
Randomization	Not a clinical trial
Blinding	Not a clinical trial

Reporting for specific materials, systems and methods

We require information from authors about some types of materials, experimental systems and methods used in many studies. Here, indicate whether each material, system or method listed is relevant to your study. If you are not sure if a list item applies to your research, read the appropriate section before selecting a response.

Materials & experimental systems

n/a	Involved in the study	
☒	☐	Antibodies
☒	☐	Eukaryotic cell lines
☒	☐	Palaeontology and archaeology
☒	☐	Animals and other organisms
☐	☒	Human research participants
☐	☒	Clinical data
☒	☐	Dual use research of concern

Methods

n/a	Involved in the study	
☒	☐	ChIP-seq
☒	☐	Flow cytometry
☒	☐	MRI-based neuroimaging

Human research participants

Policy information about studies involving human research participants

Population characteristics	Our study cohort included 516,250 children aged 3 to 5 years affiliated to the Fondo Nacional de Salud (FONASA), the public national healthcare system. 490,694 children were included in the final study population. 194,427 had received two doses of CoronaVac, 28 days apart between December 6, 2021, and February 26, 2022, and 189,523 had not received any Covid-19 vaccination by the end of the follow-up period. We excluded children who had probable or confirmed Covid-19 according to reverse-transcription polymerase-chain-reaction assay for SARS-CoV-2 or antigen test before December 6, 2021 (Figure 1). The cohort characteristics are described in Extended Data Tables 1 and 2.
Recruitment	Does not apply
Ethics oversight	The research protocol was approved by the Comité Ético Científico Clínica Alemana Universidad del Desarrollo. The study was considered exempt from informed consent.

Note that full information on the approval of the study protocol must also be provided in the manuscript.

Clinical data

Policy information about clinical studies

All manuscripts should comply with the ICMJE guidelines for publication of clinical research and a completed CONSORT checklist must be included with all submissions.

Clinical trial registration	Does not apply

Study protocol	Does not apply
Data collection	Does not apply
Outcomes	Laboratory confirmed SARS-CoV-2 infection (Covid-19), Covid-related hospitalization, Covid-related ICU admission, Covid-related death (U07.1)
